# Interscalene Block in an Anesthetized Adult with Hypertrophic Obstructive Cardiomyopathy Undergoing Clavicle Fracture Reduction 

**DOI:** 10.24908/pocus.v5i1.14223

**Published:** 2020-07-06

**Authors:** Anthony M-H Ho, Joel Parlow, Rene Allard, Michael McMullen, Glenio B Mizubuti

**Affiliations:** 1 Department of Anesthesiology and Perioperative Medicine, Queen's University Kingston, Ontario Canada

**Keywords:** Regional anesthesia, Hypertrophic Obstructive Cardiomyopathy, Interscalene Block, General anesthesia, Postoperative analgesia

## Abstract

Whether regional anesthesia procedures should be performed in heavily sedated/anesthetized adults remains controversial. One of the purported advantages of performing regional nerve blocks in conversant patients is early warning against major nerve injury and, arguably, early detection of local anesthetic systemic toxicity. A 60-year-old man with hypertrophic obstructive cardiomyopathy (HOCM) underwent a clavicle fracture repair under general anesthesia. Intraoperative transesophageal echocardiography revealed dynamic left ventricular outflow track obstruction and systolic anterior motion of the posterior mitral valve leaflet. In part based on such echo findings, he received an ultrasound-guided interscalene plus a superficial cervical plexus block for postoperative analgesia prior to emergence from general anesthesia. Given the lack of robust data on the safety of ultrasound-guided regional techniques in heavily sedated/anesthetized adults, we use the example of echographic evidence of significant HOCM to argue for a pragmatic and individualized approach when faced with unusual situations in which the pros of such an approach may outweigh the cons – in this case for performing an interscalene block on an anesthetized adult.

## Introduction

One of the purported advantages of performing regional nerve blocks in awake patients is early warning against major nerve injury and, arguably, early detection of local anesthetic systemic toxicity (LAST). For example, a needle tip positioned within the epineurium of a nerve may elicit severe paresthesia, alerting the care provider to potential intraneural injection. Specifically, interscalene block (ISB) was singled out as the one block that should be performed only in awake/lightly sedated patients, including children (Class I recommendation), in the first American Society of Regional Anesthesia (ASRA) practice advisory 1. Even though this recommendation is no longer present in the society’s second practice advisory^2^ the controversy on whether ISB should be performed in heavily sedated/anesthetized adults continues 3. 

## Case Report

A 60-yr-old man was scheduled for a right clavicle fracture internal fixation. Preoperatively, his pain level at rest was adequately managed with acetaminophen, ibuprofen, and oral hypdromorphone.

His past medical history consisted of echocardiographic evidence of hypertrophic obstructive cardiomyopathy (HOCM). The cardiac investigation was triggered by a syncopal episode when he was dehydrated. He did not normally engage in prolonged strenuous activity and had no other major co-morbidities. 

**Figure 1:**
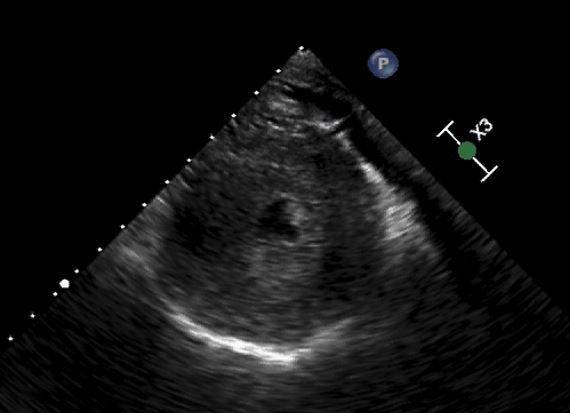
Transgastric midpapillary short axis at view at end-systole showing a severely concentric hypertrophied left ventricle.

**Figure 2:**
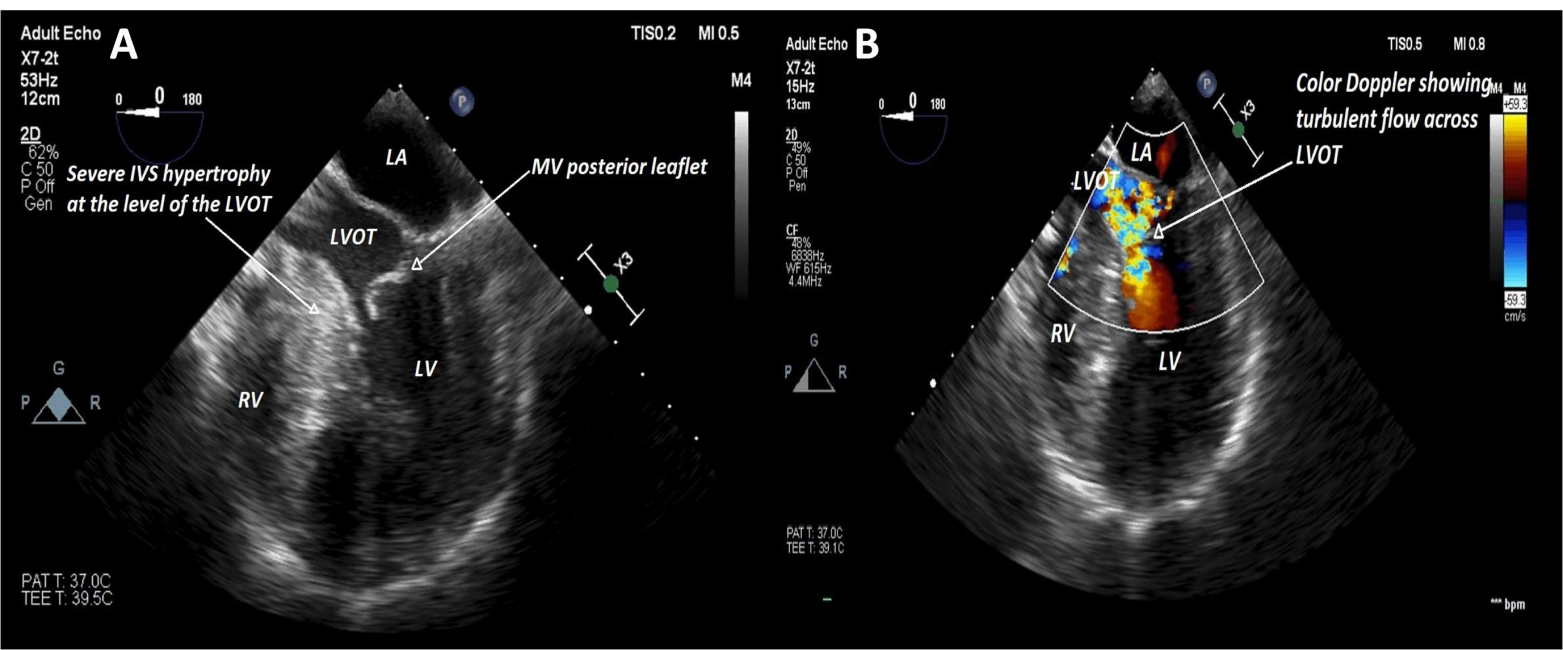
A) Midesophageal 5-chamber view at end-systole showing significant myocardial hypertrophy at the level of the LVOT, and the mitral valve posterior leaflet being drawn into the LVOT. B) Midesophageal 5-chamber view at end-systole with color Doppler showing turbulent flow through a constricted LVOT. LA = left atrium, LV = left ventricle, RV, right ventricle, LVOT = left ventricular outflow tract, IVS = interventricular septum, MV = mitral valve.

Given the proximity of the surgical site to the head, an estimated surgical time of 2½ hours, and the possibility of intraoperative breakthrough pain, we opted for general anesthesia (GA) which also allowed intraoperative transesophageal echocardiography, in addition to standard monitors and an arterial line. The patient had agreed to an interscalene block (ISB) plus a superficial cervical plexus block (SCPB) but was anxious and was reluctant to have the block while awake. Given the risk of an anxiety-driven sympathetic surge, and after discussing the risks of performing an ISB under GA, the patient was open to the idea of receiving a block prior to emergence from GA, or receiving the block after emergence from GA and if other means of pain control have failed. He was given a 500 mL-crystalloid bolus prior to induction. Perioperative hemodynamic goals included maintaining euvolemia and avoiding excessive sympathetic stimulation. Phenylephrine and esmolol were on standby. The procedure proceeded uneventfully. HOCM, systolic anterior motion (SAM) of the mitral valve leaflet, and severe concentric left ventricular hypertrophy (Figure 1) with preserved systolic function were evident on intraoperative transesophageal echocardiography. SAM was from a redundant posterior mitral leaflet moving across the left ventricular outflow tract (LVOT) during systole (Figure 2A and Video S1). Intraoperative echocardiogram found an LVOT gradient of 24 mm Hg, and colour Doppler across the LVOT demonstrated flow acceleration and turbulence associated with dynamic outflow obstruction (Figure 2B). Prior to emergence from GA, a single-shot ISB (30 mL) and SCPB (10 mL) were performed under ultrasound (US) guidance with ropivacaine 0.5%. An in-plane technique was used, and caution taken to ensure that the needle was advanced only with the tip in view, and that the injections did not require excessive pressure and were preceded by negative aspirations. The use of ultrasound provided visualization of the spread of local anesthetic in real time. The patient was then allowed to emerge and transferred to the recovery room, where he reported 0/10 pain. He had a hoarse voice but no Horner’s syndrome or dyspnea. He started to feel discomfort at the surgical site 15 hours later. From there onwards, his pain was adequately controlled with low doses of acetaminophen and hydromorphone. He was discharged the next morning and did not report any neurologic symptoms postoperatively.

## Discussion   

Anesthesiologists generally consider patient wakefulness to be a good, albeit not always reliable, monitor against nerve injury during regional anesthesia 3. At our institution, most shoulder surgeries are done under GA with an ISB performed typically before the awake/slightly sedated patient is taken to the operating room, or in the recovery room following surgery. 

In this instance, we considered the presence of hemodynamically significant HOCM (the presence of intraoperative SAM and LVOT dynamic obstruction with significant gradient under near-ideal general anesthetized and well hydrated state) justification for ISB and SCPB under GA. In HOCM, the higher velocity of blood travelling across a hypertrophied LVOT generates a Venturi effect that may draw a floppy mitral valve leaflet into the LVOT, exacerbating the dynamic obstruction. The vast majority of SAM involves the anterior leaflet, only occasionally the posterior leaflet, as in our patient. The loss of mitral valve leaflet apposition usually leads to mitral regurgitation, further compromising forward flow. SAM can be triggered by reduced ventricular filling and/or exaggerated ventricular emptying 4. Unexpected perioperative circulatory deterioration can occur in the setting of HOCM 4; and cardiac events are common in patients with septal hypertrophy 5. In patients with HOCM, maintenance of adequate cardiac preload and avoiding increased sympathetic stimulation are cornerstones of perioperative management. In our patient, the pain level at rest from his fracture was low before surgery, and performing the blocks awake in an anxious and reluctant patient could potentially trigger a sympathetic surge. During nerve blocks in anxious patients, a vasovagal response is not uncommon, which may mimic circulatory collapse in HOCM. Furthermore, several extra hours of postoperative analgesia could be gained by blocking at the end of the case. Performing the regional blocks after the patient had emerged from GA would increase the risk of severe postoperative pain and associated sympathetic stimulation on the one hand. A heavily narcotized patient, on the other hand, may have CO_2_ retention with increased catecholamine output and may be an unreliable monitor against nerve injury and LAST, may have airway obstruction, and may not be able to maintain adequate positioning for the block. Furthermore, a decision had to be made at the end of the case whether surgeon administered local infiltration should be used. From experience, local infiltration of the operative field by surgeon is not very effective; it also reduces the amount of LA that could be used during a subsequent rescue nerve block. As a result, performing the US-guided blocks under GA in our case appeared to be prudent after detailed consideration of the risks and benefits.

The dated ASRA Class I recommendation for ISB to be performed only in awake/lightly sedated patients was based in part on case reports, including 4 patients with spinal cord injuries who had ISB while under GA 6. Three of those cases were guided by electrical stimulation and 1 patient’s block was performed by walking the needle tip off the C6 transverse process 6. A case of inadvertent intrathecal placement of an interscalene catheter has also been described 7: A Tuohy needle was inserted using landmark technique guided by electrical simulation while the patient was unconscious; subsequent intrathecal injection of bupivacaine via a catheter which had been threaded 7 cm beyond the Tuohy tip led to death. In such cases, it was assumed that had the patients been awake during the block or catheterization, pain would have been elicited. This assumption is reasonable, as other authors reporting cases of inadvertent subarachnoid/subdural injections during non-US guided ISB in wakeful patients had all reported paresthesia or severe pain 8. Although no subarachnoid injection has apparently appeared in the literature using US in ISB in anesthetized patients, it can occur if the operator loses track of the tip of the block needle. Fortunately, visualizing the entire needle is easy in ISB and SCPB using an in-plane approach because of the near-orthogonal orientation between the needle and the US beam. As for the argument that a wakeful patient might reduce the risk of intraneuronal injection, the intuitive answer is ‘yes’ but there is no experimental data to confirm this assumption. Given the low incidence of serious nerve injury 9, and the general acceptance of blocks in conversant adults, such data are unlikely to become available. Whether to block someone awake or anesthetized must therefore be considered on an individual basis, and one must not be dogmatic by shutting the door on any option. In weighing the pros/cons, the following factors need to be considered. First, the incidence of major nerve injury and LAST after ISB in anesthetized children (mostly US-guided and between 10-18 years of age) is low (0/518 ISBs) 10. Second, postoperative neurologic symptoms (PONS) have occurred after blocks in wakeful patients who did not report paresthesia 1. Indeed, Perlas et al. have found that paresthesia in wakeful patients was reported in only 38% of needle-to-nerve contacts in axillary blocks 11, bearing in mind that needle-to-nerve contact is less traumatic than intraneuronal injection. Third, not all paresthesia leads to PONS 12. For example, Candido et al. prospectively collected PONS data after 693 ISBs in conversant patients using nerve stimulation 12. There were 29 patients with 31 post-ISB PONS that lasted up to several months, but only 7 of those were blocks in which paresthesia was elicited 12. Fourth, current US probes lack the resolution to detect intraneuronal injury 13, and the operator must rely on injection pressure and current intensity to help prevent nerve injury. Fifth, the overall success rate of ISB in anesthetized patients in experienced hands using nerve stimulation alone was 97%; PONS occurred in 4.4% (40 of 910) of patients, and long-term neurologic complications occurred in 0.8% (8 of 910) 14. One might expect similar results from US-guided ISB as a randomized controlled trial comparing US- and nerve stimulation-guided ISB had observed no significant differences in block failures, patient satisfaction, or incidence and severity of PONS 15.

## Conclusion

 Given the lack of robust data on the safety of US-guided ISB in anesthetized patient, we use the example of symptomatic HOCM to argue for a pragmatic and individualized approach when faced with unusual situations in which the pros of such an approach may outweigh the cons.

## Declaration of patient consent 

The authors certify that they have obtained all appropriate patient consent forms. In the form the patient(s) has/have given his/her/their consent for his/ her/their images and other clinical information to be reported in the journal. The patients understand that their names and initials will not be published and due efforts will be made to conceal their identity, but anonymity cannot be guaranteed. This study was approved by the Queen’s University Health Sciences and Affiliated Teaching Hospitals Research Ethics Board. 

## Disclosures

None

## Supplementary Material

Video S1 Midesophageal 5-chamber view showing a redundant posterior mitral valve leaflet moving across the LVOT during systole. Note the severely hypertrophied interventricular septum, especially at the mid and basal levels.
